# Interruption time series analysis using autoregressive integrated moving average model: evaluating the impact of COVID-19 on the epidemic trend of gonorrhea in China

**DOI:** 10.1186/s12889-023-16953-5

**Published:** 2023-10-23

**Authors:** Yanyan Li, Xingyan Liu, Xinxiao Li, Chenlu Xue, Bingjie Zhang, Yongbin Wang

**Affiliations:** Department of Epidemiology and Health Statistics, School of Public Health, The First Affiliated Hospital, Xinxiang Medical University, Xinxiang, 453000 Henan Province People’s Republic of China

**Keywords:** Autoregressive integrated moving average models, Interrupted time series analysis, Intervention analysis, COVID-19, Gonorrhea

## Abstract

**Background:**

Interrupted time series (ITS) analysis is a growing method for assessing intervention impacts on diseases. However, it remains unstudied how the COVID-19 outbreak impacts gonorrhea. This study aimed to evaluate the effect of COVID-19 on gonorrhea and predict gonorrhea epidemics using the ITS-autoregressive integrated moving average (ARIMA) model.

**Methods:**

The number of gonorrhea cases reported in China from January 2005 to September 2022 was collected. Statistical descriptions were applied to indicate the overall epidemiological characteristics of the data, and then the ITS-ARIMA was established. Additionally, we compared the forecasting abilities of ITS-ARIMA with Bayesian structural time series (BSTS), and discussed the model selection process, transfer function, check model fitting, and interpretation of results.

**Result:**

During 2005–2022, the total cases of gonorrhea were 2,165,048, with an annual average incidence rate of 8.99 per 100,000 people. The highest incidence rate was 14.2 per 100,000 people in 2005 and the lowest was 6.9 per 100,000 people in 2012. The optimal model was ARIMA (0,1, (1,3)) (0,1,1)_12_ (Akaike’s information criterion = 3293.93). When predicting the gonorrhea incidence, the mean absolute percentage error under the ARIMA (16.45%) was smaller than that under the BSTS (22.48%). The study found a 62.4% reduction in gonorrhea during the first-level response, a 46.47% reduction during the second-level response, and an increase of 3.6% during the third-level response. The final model estimated a step change of − 2171 (95% confidence interval [*CI*] − 3698 to − 644) cases and an impulse change of − 1359 (95% *CI* − 2381 to − 338) cases. Using the ITS-ARIMA to evaluate the effect of COVID-19 on gonorrhea, the gonorrhea incidence showed a temporary decline before rebounding to pre-COVID-19 levels in China.

**Conclusion:**

ITS analysis is a valuable tool for gauging intervention effectiveness, providing flexibility in modelling various impacts. The ITS-ARIMA model can adeptly explain potential trends, autocorrelation, and seasonality. Gonorrhea, marked by periodicity and seasonality, exhibited a downward trend under the influence of COVID-19 intervention. The ITS-ARIMA outperformed the BSTS, offering superior predictive capabilities for the gonorrhea incidence trend in China.

**Supplementary Information:**

The online version contains supplementary material available at 10.1186/s12889-023-16953-5.

## Background

Gonorrhea, caused by *Neisseria gonorrhoeae*, is a sexually transmitted disease leading to purulent infections in the urinary and reproductive systems. It is estimated that approximately 86.9 million adults develop the illness annually [1]. In men, it may present as urethritis, and in women, as cervicitis or urethritis, affecting various genital sites (pharynx, rectum, and conjunctiva) [2, 3]. In recent years, there has been a significant global increase in gonorrhea cases. In China, since its resurgence in 1975, the number of patients has consistently risen each year. Although there was a temporary decline due to an increase in syphilis cases, gonorrhea remains a prevalent sexually transmitted disease in China, classified as a Class B infectious disease according to the Law of the People’s Republic of China on the Prevention and Treatment of Infectious Diseases. In 2019, gonorrhea ranked fourth among the reported Class A and B infectious diseases in China and continued to be a major contributor to the overall infectious disease burden. Gonorrhea not only poses direct health risks but also increases the transmission and acquisition of other sexually transmitted infections, including HIV. During pregnancy, *Neisseria gonorrhoeae* infections raise concerns as infected pregnant women can transmit the bacterium to the fetus during childbirth, leading to neonatal ophthalmia. This highlights the substantial public health and socioeconomic consequences of gonorrhea globally [4]. Gonorrhea stands as the second most common bacterial sexually transmitted infection today. Despite its typically uncomplicated clinical progression, it can lead to severe complications such as salpingitis, ectopic pregnancy, infertility, prostatitis, gonococcal conjunctivitis, and disseminated gonococcal infection [5–7]. Furthermore, the cardiovascular and nervous system may also be involved [5–7].

The World Health Organization (WHO) had declared COVID-19 a global emergency on January 30, 2020. The causative agent, severe acute respiratory syndrome coronavirus 2 (SARS-CoV-2), can lead to respiratory diseases, pneumonia, lung failure, and death [8]. This pandemic has exerted immense pressure on global medical systems, as early reports from China underscored the strain on hospital staff [9]. Governments worldwide implemented diverse policies and restrictive measures to reduce the spread of COVID-19. Despite an initial decline in reported disease transmission during the epidemic blockade, there was a rebound by the year’s end [10]. The impact of the COVID-19 pandemic extends beyond its direct effects, potentially causing delays in detecting sexually transmitted infections and accessing medical care. This poses challenges for individuals in identifying conditions like gonorrhea promptly, leading to postponed treatment and increased health risks [10]. While combating COVID-19, studies indicated a decrease in the incidence of respiratory and intestinal infectious diseases in 2020 [11], likely attributed to the reallocation of medical resources and interruptions in non-COVID-19 medical services. Consequently, treatment delays for sexually transmitted diseases occurred, with individuals resorting to inappropriate self-treatment or remaining untreated post-infection. It is speculated that implementing social, physical, and travel restrictions, coupled with recommendations for hand disinfectants and mask-wearing, may contribute to diminishing the spread of general infectious diseases. Regarding the effectiveness of the COVID-19 pandemic on sexually transmitted infections, intriguingly, some studies indicated a low severity index only in the initial months of the pandemic. However, it is premature to assess the long-term impact on gonorrhea incidence during the early stages of the COVID-19 pandemic, necessitating further research to explore additional factors influencing sexual behavior during the ongoing COVID-19 situation [12].

Interrupted time series (ITS) analysis is a powerful and increasingly popular design for evaluating public health interventions. This approach estimates trend changes in comparison to a counterfactual scenario after the intervention. In the absence of the intervention, the counterfactual represents the anticipated continuous trend. The analysis categorizes time into “before the intervention” and “after the intervention” stages, offering a valuable comparison to assess intervention impacts by scrutinizing changes during the intervention period. In our study, we employed ITS analysis to evaluate the impact of COVID-19 on gonorrhea incidence both before and after its occurrence. The ITS-autoregressive integrated moving average (ARIMA) model was also utilized to predict the trend of gonorrhea incidence in China. This predictive model serves as a foundation for informing strategies in the prevention and control of gonorrhea.

## Materials and methods

Monthly gonorrhea case notifications spanning January 2005 to September 2022 were obtained from the National Notifiable Infection Disease Surveillance System (NNIDSS). Simultaneously, population data were extracted from the Statistical Yearbook of China. A total of 18 years of data were collated for the analysis of this study. Subsequently, we constructed the ITS-ARIMA model using the data between January 2005 and December 2019 to forecast the number of gonorrhea cases between January 2020 and September 2022. This approach facilitated the evaluation of disparities between the predictions and the actual values, offering a comprehensive assessment of the intervention’s effectiveness. Furthermore, we conducted a sensitivity analysis using the Bayesian structural time series (BSTS) to affirm the robustness of the ITS-ARIMA.

Numerous statistical models can be employed for ITS analysis [13]. Currently, the two most popular models are the ARIMA and Segmented Regression (SR) models. For effective ITS analysis, it is essential that the changing trend of the dependent variable before and after the intervention follows a linear pattern, making SR model a suitable approach for such cases. However, if the time series data exhibits a non-linear trend, potential seasonality, or periodicity, ITS-ARIMA would become a valuable tool. ITS-ARIMA model needs to be established through a series of procedures to explain the autoregression, moving average, stationarity, and other characteristics of the time series.

ITS analysis involves collecting data at various time points both before and after the intervention, aiming to mitigate the influence of any pre-existing declining or rising trends in the outcome variable. To comprehensively assess the impact of the intervention, statistical models are deployed. These models evaluate changes in both the level and trajectory of the outcome variable before and after the intervention point. The ITS design involves scrutinizing trends in the variables of interest and estimating deviations from what would have been expected in the absence of the intervention, often referred to as the counterfactual trend.

### Fitting ARIMA model

**Fig. 1:**
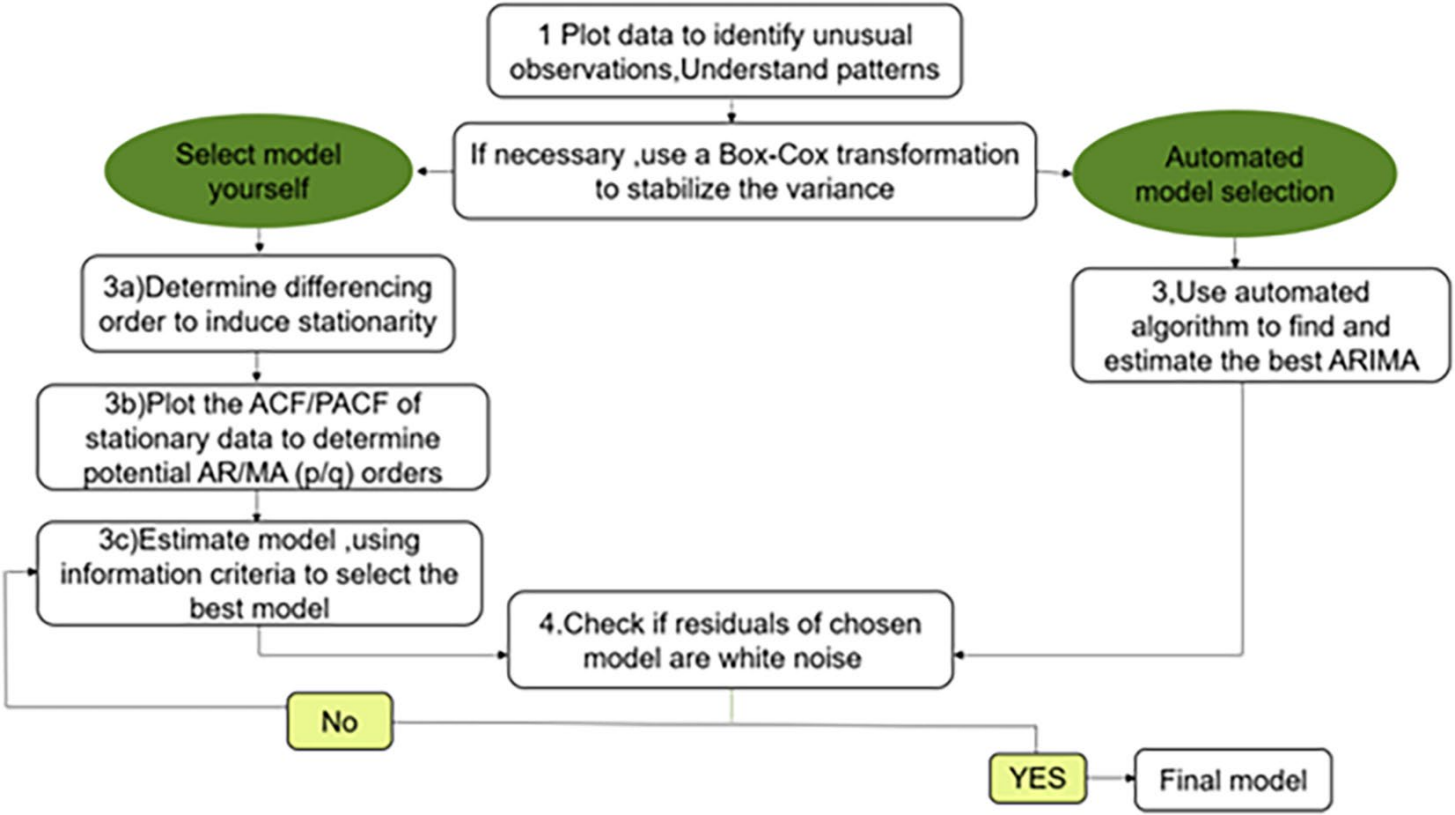
Flow chat for ARIMA model selection

ARIMA model is a famous time series prediction method proposed by Box and Jenkins [14, 15]. ARIMA (*p*, *d*, *q*) stands for the autoregressive integrated moving average model. In this acronym, AR represents autoregressive, denoted by p which signifies the autoregressive order. MA represents moving average, indicated by q representing the number of moving average terms. The d in ARIMA refers to the order of differencing applied to stabilize the time series data before modelling, ensuring that it becomes stationary [16]. All the parameters of the ARIMA could be determined through the three iterative steps of model identification, parameter selection, and model verification [17–19] (Fig. 1).

**(1) Model stationarity test:** An autocorrelation function (ACF) plot illustrates the correlation between each observed data point and its preceding value at various lags. When seasonality is evident in time series data, it is commonly addressed by implementing a seasonal differencing process within the ARIMA model **(2) Model selection:** In our research, we used the automated algorithm, auto.arima(), to identify suitable ARIMA model. This algorithm, available in the R package, has the ability to pinpoint potential values for the p and q parameters. Nevertheless, there are instances where these parameters may be estimated by examining ACF and Partial ACF (PACF) plots. To ensure a good fit, we relied on two crucial information criteria: Akaike’s information criterion (AIC) and Bayesian information criterion (BIC). The optimal ARIMA model was identified by minimizing these two information criteria **(3) Model checking:** This stage mainly tests whether the fitted model is reasonable. It involves two key assessments: first, testing the significance of the estimated values of model parameters, and second, examining whether the residual series of the model demonstrates characteristics of white noise. Model verification is essential to confirm how well the model aligns with the data. If the model effectively captures the underlying correlations, the residuals from the model should behave like a white noise series. This evaluation was carried out by scrutinizing residual plots and conducting tests such as the Ljung-Box Q test for residuals.

### Use the ITS-ARIMA to evaluate interventions

ITS analysis is employed to assess the impact of intervention implementation on the observed outcomes, referred to as the “intervention effect”. We conducted a comparison between the pre-intervention and the post-intervention to evaluate whether there was a significant change in the post-intervention compared to the pre-intervention [20]. Although various effects could be observed, we focused on three main types: step change, pulse, and ramp [17]. If we used T_0_ to represent the starting time of the intervention, these effects would be summarized as follows:

1. **Step change:** A sudden and sustained change where the time series is shifted either up or down by a given value immediately following the intervention. The step change variable takes the value of 0 before the start of the intervention, and 1 afterward.
2. **Pulse:** A sudden and temporary change that is observed for one or more time points immediately after the intervention, followed by a return to the baseline level. The pulse variable takes the value of 1 on the date of the intervention, and 0 otherwise.a
3. **Ramp:** A change in slope that occurs immediately after the intervention. The ramp variable takes the value of 0 before the start of the intervention and increases by 1 after the date of the intervention.

### Transfer functions

We assumed that the most likely response of COVID-19 to gonorrhea would include one or more of the following combinations: (1) Transient changes followed by a return to the previous level; (2) Long term changes in levels; (3) Changes in the time series characterized by a ramp. These modes are represented by combinations or slopes of step functions and impulse functions. All trends are assumed to be “0” before the starting point of COVID-19. Subsequently, the model and transfer function selection were estimated based on the cross-correlation function between the assumed function and the time series, assuming that the transfer function is only applied at the beginning of the delay function or its shape undergoes a brief modification. The optimal model is chosen by considering criteria such as the minimum AIC, prediction variance, the number of effective items, and simplicity [21]. The ARIMA model can extend beyond the basic intervention influence shape, incorporating more complex effects through the “transfer function”. This function describes the relationship between the intervention (occurrence of COVID-19) and the outcome (gonorrhea incidence), modifying the relationship between different types of changes (step change, pulse, and ramp) and time series to fit a more intricate relationship. Model fitting statistics, such as AIC or BIC, aid in determining the most suitable form of the transfer function and the timing of the event.

## Results

### Statistical description

**Fig. 2:**
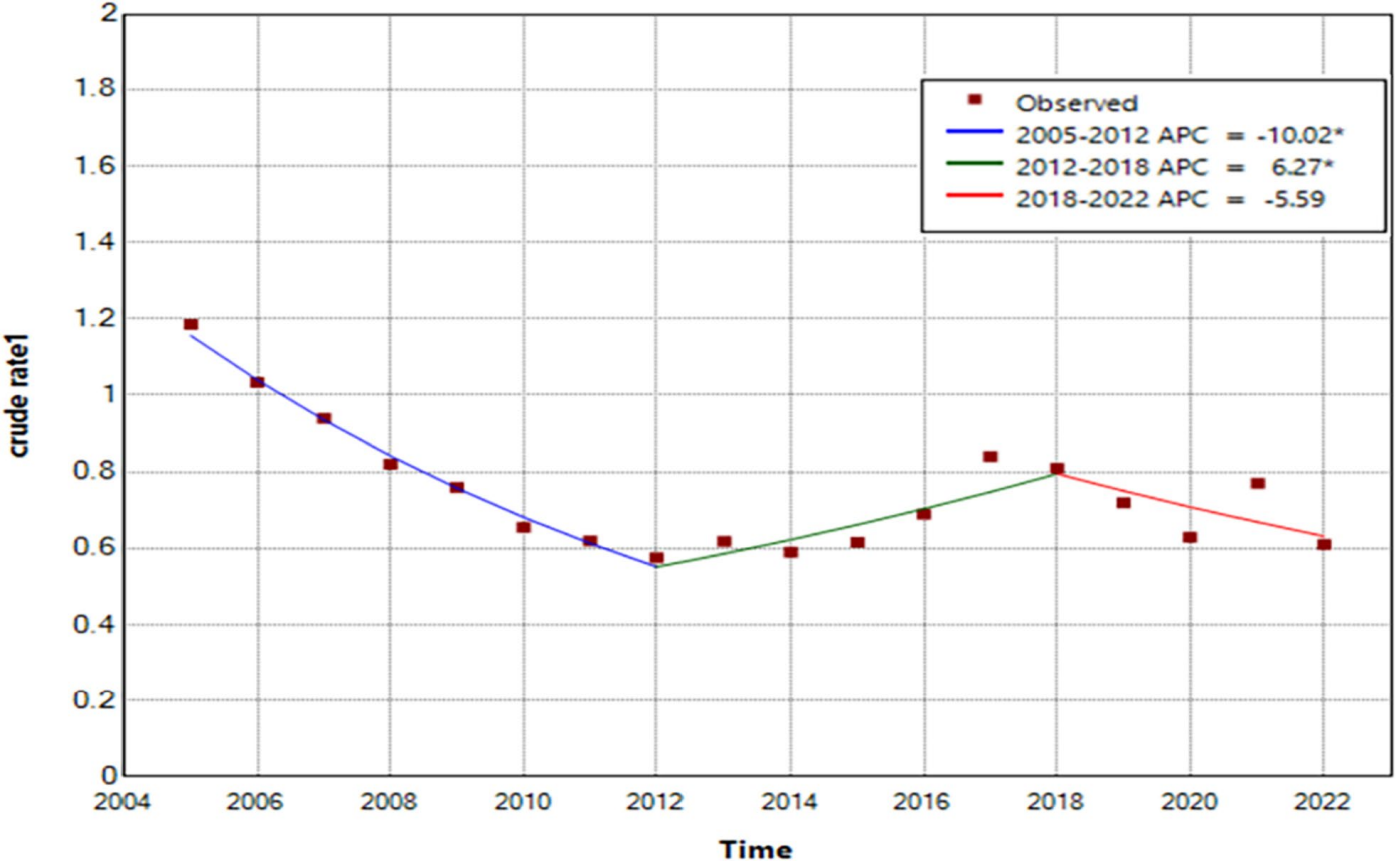
Joinpoint regression plot displaying the gonorrhea epidemiological trends from 2005 to 2022

During 2005–2022, the total number of reported gonorrhea cases was 2,165,048, with an annual average incidence rate of 8.99 per 100,000 people. The highest incidence rate, observed in 2005, was 14.2 per 100,000 people, while the lowest occurred in 2012 at 6.9 per 100,000 people. Overall, a declining trend was evident in the reported gonorrhea cases, with an average annual percentage change (AAPC) of -3.5 (95% confidence interval [*CI*] -5.9 to -1.0; *t* = -6.818, *P* = 0.006). The trend exhibited three stages: a rapid reduction from 2005 to 2012, with an annual percentage change (APC) of-10.0 (95% *CI* -12.8 to -7.1; *t* = -7.4, *P* < 0.001); a rapid rise from 2012 to 2018 (APC = 6.3, 95% *CI* 0.8 to 12.1; *t* = 2.5, *P* = 0.029); and a slight reduction from 2018 to 2022 (APC = -5.6, 95% *CI* -12.4 to 1.8; *t* = -1.7, *P* = 0.12) (Fig. 2). The seasonal indexes of gonorrhea incidence data from January to December were 0.94, 0.74, 0.94, 0.95, 1.01, 1.04, 1.09, 1.09,1.06, 1.02, 1.07, and 1.08, respectively. This indicated periodic and seasonal fluctuations in gonorrhea incidence, with higher incidence observed in July and August and lower incidence in February each year.

### Plot data

**Fig. 3:**
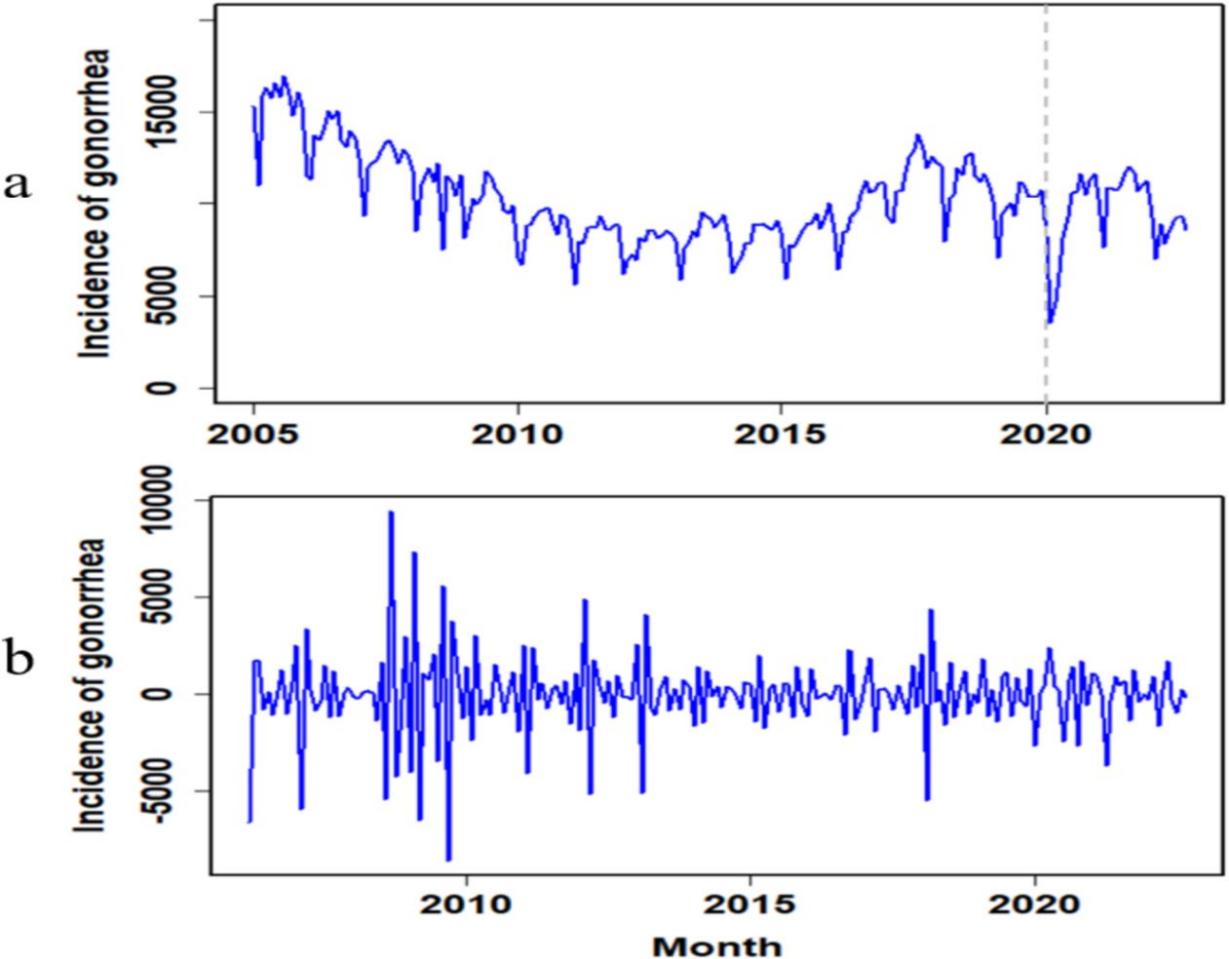
Monthly gonorrhea incidence series and the differenced series. **a** Time series plot showing the original gonorrhea incidence; (**b**) Time series plot showing the seasonally and nonseasonally differenced gonorrhea incidence

The incidence data of gonorrhea was modelled using ITS-ARIMA. In Fig. 3, the monthly incidence time series of gonorrhea in China was depicted from January 2005 to September 2022. The gonorrhea incidence series before the COVID-19 intervention spanned from January 2005 to December 2019, while that after the COVID-19 intervention spanned from January 2020 to September 2022. As shown in Fig. 3a, gonorrhea exhibited a pronounced downward trend in the initial stages of the COVID-19 outbreak in 2020, followed by an upward trend. Notably, there were evident periodic and seasonal patterns between January 2005 and September 2022. After seasonally and nonseasonally differencing once, the time series plot is provided in Fig. 3b.

### Select model

**Fig. 4:**
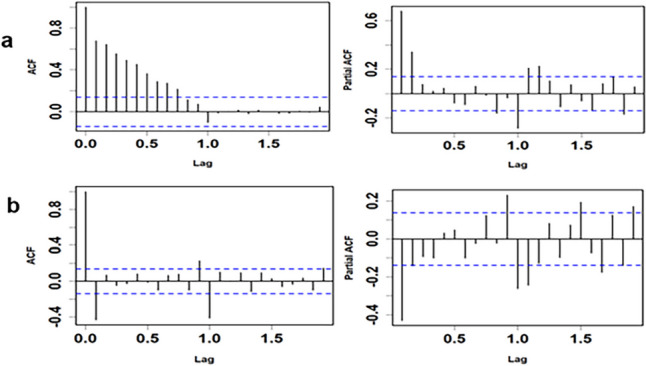
Autocorrelation and partial autocorrelation function (ACF and PACF) plots. **a** Seasonal differencing; (**b**) nonseasonal differencing

Given the presence of seasonality, we first performed a seasonal and nonseasonal difference. After mitigating both trend and seasonality, the gonorrhea time series tends toward stability. In the ACF and PACF plots (Fig. 4), bars above or below the dotted line indicated statistically significant autocorrelation (*P* < 0.05). Upon applying seasonal differencing to the raw data, noticeable autocorrelations emerged in the ACF plot (Fig. 4a). A comparison between Fig. 4a and b revealed that most of the autocorrelations were effectively eliminated through only the first-order difference.

### Check residuals

The optimal model was chosen based on minimizing both AIC and prediction variance. To identify ITS-ARIMA model components, we employed the automated algorithm auto.arima() from the prediction package in R. This algorithm iteratively explores potential ARIMA models to find the one with the lowest AIC or BIC.

**Fig. 5:**
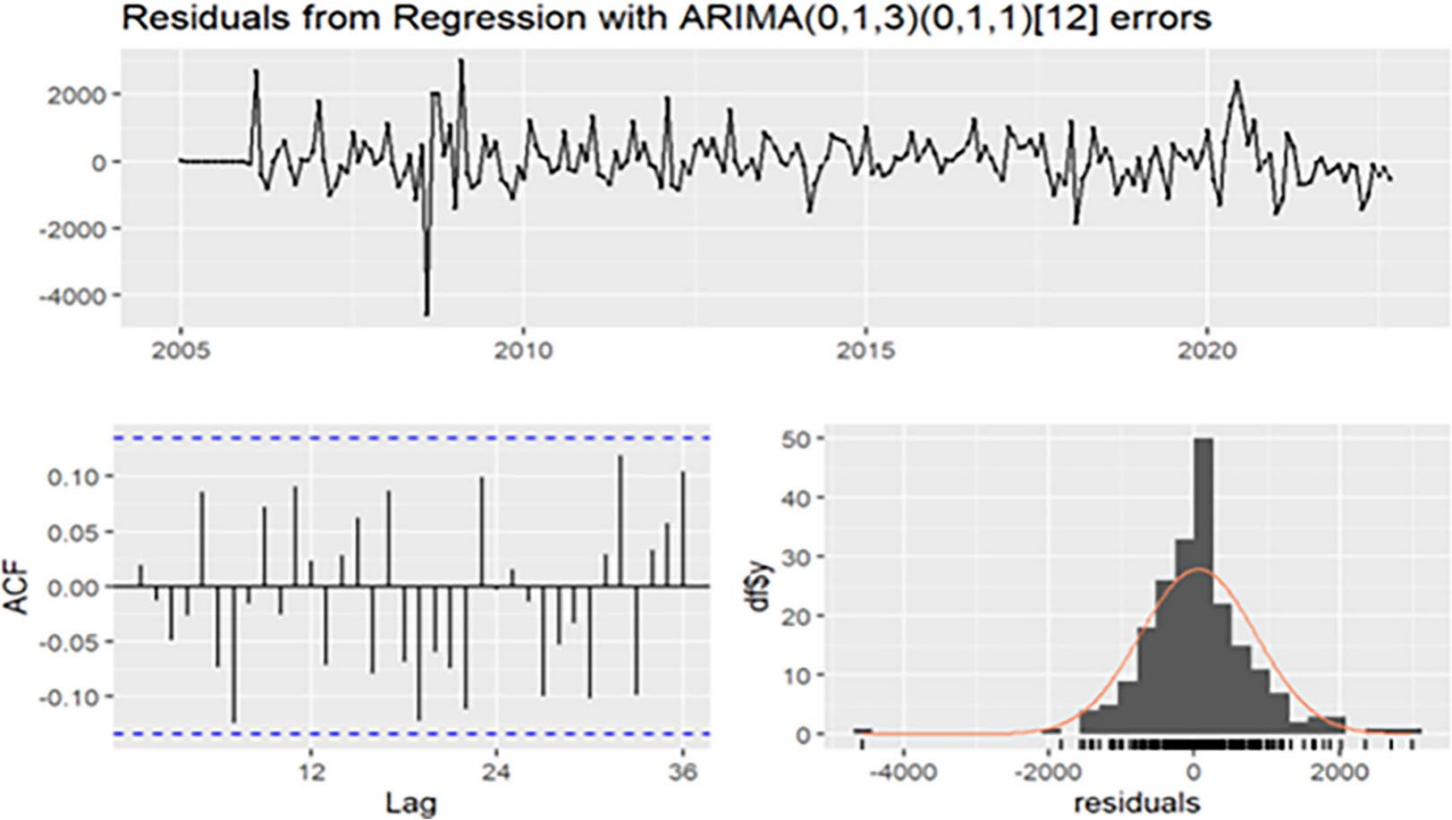
Residual check for the final ARIMA (0,1,3)(0,1,1)_12_ model

The “auto. arima” program was used to simulate the gonorrhea epidemic data from January 2005 to September 2022. Following the program run, the ARIMA (0,1,3)(0,1,1)_12_ (AIC = 3278, AICc = 3278.58, and BIC = 3301.09) was initially selected through step and pulse changes, but MA2 = 0.098 (*t* = 1.150, *P* = 0.125) did not pass the test, leading to the deletion of this coefficient. The ARIMA(2,1,0)(1,1,1)_12_ model (AIC = 3286.11, AICc = 3286.69, BIC = 3309.19, and LL = -1636.05) was selected through step and ramp changes. Considering that the ARIMA (0,1,3)(0,1,1)_12_ model with the step and pulse changes presented lower values of AIC and BIC (Table 1), the sparse coefficient ARIMA (0,1, (1,3)) (0,1,1)_12_ (MA1 = -0.62, *t* = -8.63, *P* < 0.01; MA3 = 0.157, *t* = 1.86, *P* < 0.05, and SMA1 = -0.7996, *t* = -12.90, *P* < 0.001) was thus selected as the optimal model. Subsequently, residual checkes are provided in Fig. 5, suggesting a roughly constant variance as time increases. The histogram indicated that prediction errors were approximately normally distributed with a mean close to 0. No evident pattern or significant autocorrelation was observed in the residuals, supporting the normal distribution. The *P*-value for Ljung-Box Q test was 0.062, showing a white noise series of the forecast residuals. These results indicated a good fit of the chosen model.

**Table 1:** Identified possible ITS-ARIMA with the AIC, AICc, BIC, and LL

ARIMA model	AIC	AICc	BIC	LL
ARIMA(0,1,3)(0,1,1)_12_	3278	3278.58	3301.09	-1632
ARIMA(1,1,2)(0,1,1)_12_	3279.25	3279.83	3302.34	-1632.63
ARIMA(2,1,1)(0,1,1)_12_	3280.38	3280.96	3303.46	-1633.19
ARIMA(2,1,0)(0,1,2)_12_	3280.4	3280.98	3303.49	-1633.2
ARIMA(0,1,2)(0,1,2)_12_	3281.18	3281.76	3304.27	-1633.59
ARIMA(2,1,0)(1,1,1)_12_	3286.11	3286.69	3309.19	-1636.05

The training set, consisting of data from January 2005 to December 2019, was used to derive the optimal ARIMA (0,1,3)(0,1,1)_12_ and BSTS models. Subsequently, predictions were made for the data spanning January 2020 to September 2022 (Table 2). The analysis revealed that the resulting mean absolute percentage error (MAPE) was smaller for the ITS-ARIMA model (MAPE = 16.45%) compared to the BSTS model (MAPE = 22.48%). This suggested that the forecasts generated by the ITS-ARIMA model were closer to the observed values.

**Table 2:** Prediction of gonorrhea incidence from January 2020 to September 2022 after COVID-19 intervention using the ITS-ARIMA model and BSTS model

Time	Actual value	ITS-ARIMA model		BSTS model	
		Forecasts	95% *CI*	Forecasts	95% *CI*
2020-1	8254	9489	7899 ~ 11,078	7264	5704 ~ 8969
2020-2	3524	7140	5507 ~ 8773	9355	7578 ~ 10,980
2020-3	4664	9167	7411 ~ 10,924	9454	7622 ~ 11,197
2020-4	6267	9331	7418 ~ 11,245	10,316	8519 ~ 11,994
2020-5	8104	10,077	8019 ~ 12,135	10,117	7957 ~ 12,108
2020-6	9292	10,023	7830 ~ 12,216	11,025	8820 ~ 13,447
2020-7	10,621	11,047	8727 ~ 13,368	11,050	8762 ~ 13,311
2020-8	10,724	11,193	8752 ~ 13,635	10,430	7814 ~ 13,111
2020-9	11,643	10,601	8045 ~ 13,157	10,075	7030 ~ 12,918
2020-10	10,551	10,261	7595 ~ 12,928	10,443	7365 ~ 12,993
2020-11	11,260	10,603	7831 ~ 13,375	10,298	7343 ~ 13,615
2020-12	11,691	10,621	7747 ~ 13,495	9379	6192 ~ 12,920
2021-1	10,284	9479	6377 ~ 12,580	6950	3084 ~ 10,688
2021-2	7650	7095	3878 ~ 10,312	9127	5505 ~ 12,598
2021-3	10,878	9107	5760 ~ 12,454	9188	4881 ~ 13,160
2021-4	10,874	9271	5791 ~ 12,751	10,051	5938 ~ 14,066
2021-5	10,773	10,017	6409 ~ 13,625	9874	5662 ~ 14,106
2021-6	10,950	9962	6230 ~ 13,694	10,768	6469 ~ 15,272
2021-7	11,747	10,987	7135 ~ 14,838	10,745	6267 ~ 15,612
2021-8	12,019	11,133	7165 ~ 15,101	10,195	5640 ~ 15,056
2021-9	11,744	10,541	6460 ~ 14,621	9958	5210 ~ 15,085
2021-10	10,720	10,201	6010 ~ 14,391	10,278	5153 ~ 15,468
2021-11	11,119	10,542	6245 ~ 14,840	10,204	4829 ~ 15,765
2021-12	11,264	10,560	6159 ~ 14,962	9219	3463 ~ 15,042
2022-1	9273	9418	4811 ~ 14,025	6790	1376 ~ 13,164
2022-2	6979	7035	2310 ~ 11,760	8925	3288 ~ 15,081
2022-3	8886	9047	4191 ~ 13,902	9019	2812 ~ 15,220
2022-4	7821	9210	4221 ~ 14,200	9819	3655 ~ 16,446
2022-5	8395	9956	4837 ~ 15,076	9753	2945 ~ 16,849
2022-6	8988	9902	4655 ~ 15,148	10,607	4031 ~ 17,916
2022-7	9263	10,926	5556 ~ 16,297	10,473	3160 ~ 17,568
2022-8	9275	11,073	5581 ~ 16,564	9983	3017 ~ 17,287
2022-9	8598	10,480	4870 ~ 16,090	9715	2262 ~ 17,573

**Fig. 6:**
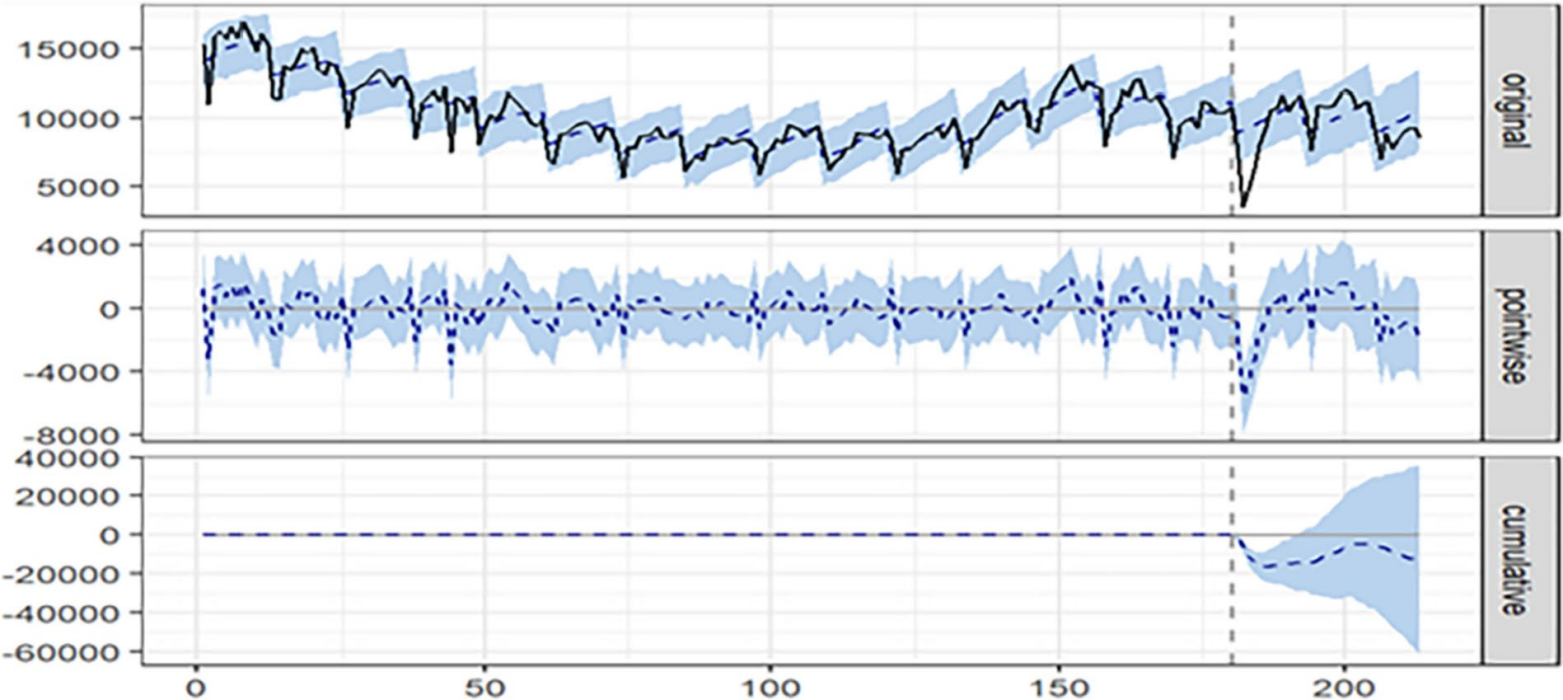
Time series plot illustrating the estimated causal effects of the COVID-19 outbreak on the decline in gonorrhea case notifications from January 2020 to September 2022

In the BSTS analysis (Fig. 6), the first panel illustrates gonorrhea case notifications alongside counterfactual forecasted results for the post-outbreak period. The second panel depicts the pointwise causal effect, indicating the disparity between actual values and forecasted values. The third panel presents the cumulative effect of the COVID-19 outbreak by aggregating the pointwise contributions from the second panel. The cumulative effect revealed that following the COVID-19 pandemic, although there was an upward trend in the later stage of gonorrhea, the overall trend remained downward. This aligned with the findings of the ITS-ARIMA model, affirming the model’s effectiveness.

Considering different national response levels, the responses were broadly categorized into three levels in out study: the first level from February 2020, the second level from March to April 2020, and the third level from May 2020 to September 2022. A comparison of predicted and actual values of gonorrhea at different response levels from February 2020 to September 2022 using the ITS-ARIMA model is presented in Table 3, revealing a 62.4% reduction in gonorrhea during the first-level response, a 46.47% reduction during the second-level response, and a 3.6% increase during the third-level response.

**Table 3:** Comparison of the forecasted and actual values at various response levels using the ITS-ARIMA model from February 2020 to September 2022

Time	True values	Predict values	Absolute effect	Relative effect (%)
2020-2	3524	9374	-5850	-62.4%
2020-3 ~ 2020-4	10,931	20,419	-9488	-46.47%
2020-5 ~ 2022-9	291,386	281,367	10,019	3.6%
2020-2 ~ 2022-9	305,841	299,786	6055	2.02%

### Final model

**Fig. 7:**
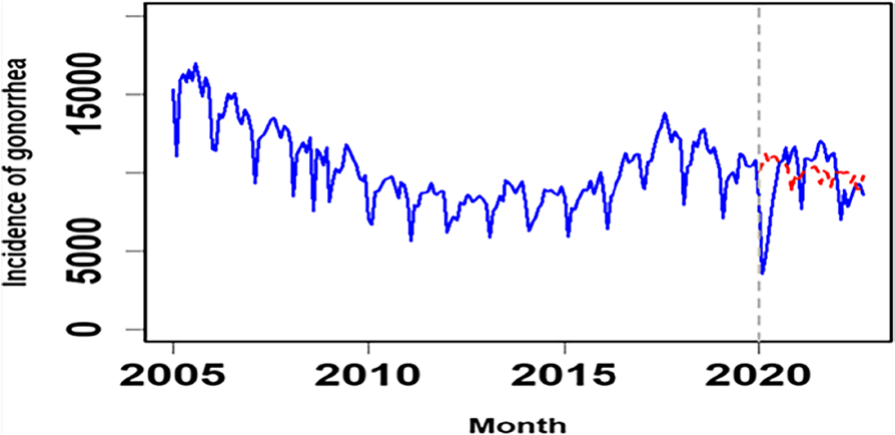
Actual values and forecasted values in the absence of intervention under the ITS-ARIMA model

After the occurrence of COVID-19, the fitting and observational values are illustrated in Fig. 7. It was projected that gonorrhea cases would decrease in January 2020, indicating a transient impact modelled as a pulse function. Post-COVID-19, gonorrhea exhibited a declining trend, suggesting potential long-term changes through a step function. The final model indicated a sudden decrease in gonorrhea cases after COVID-19, followed by a gradual return to pre-COVID-19 levels. The model assumed an immediate decrease (step change) and a pulse change in gonorrhea incidence after the intervention. The estimated final model suggested a step change of -2171 (95% CI -3698 to -644) cases and an impulse change of -1359 (95% CI -2381 to -338) cases. Gonorrhea incidence showed a declining trend in the months post-COVID-19, returning to pre-COVID-19 levels from the second half of the year to the end. Figure 7 provides a comparison of the predicted and observed values of the ITS-ARIMA model without intervention (counterfactual). Based on the ITS-ARIMA model, a re-simulation of the data from January 2005 to September 2022 predicted the gonorrhea epidemic trends in China from October 2022 to December 2023 (Table 4), estimating a total of 116,035 (95% *CI* 72,261 ~ 159,809) cases and a monthly average of 7736 (95% *CI* 4817 ~ 10,654) cases.

**Table 4:** Predicted gonorrhea incidence from October 2022 to December 2023 using the ITS-ARIMA model

Time	Forecasts	95% *CI*
2022-10	8291	6582 ~ 10,000
2022-11	8662	6776 ~ 10,548
2022-12	8768	6671 ~ 10,865
2023-01	7213	4916 ~ 9510
2023-02	4623	2142 ~ 7103
2023-03	6670	4019 ~ 9322
2023-04	6744	3931 ~ 9556
2023-05	7450	4485 ~ 10,414
2023-06	7749	4640 ~ 10,858
2023-07	8557	5310 ~ 11,805
2023-08	8658	5278 ~ 12,038
2023-09	8339	4831 ~ 11,846
2023-10	7793	4070 ~ 11,516
2023-11	8203	4326 ~ 12,081
2023-12	8315	4284 ~ 12,347

## Discussion

During the COVID-19 outbreak, all provinces in China implemented emergency responses, employing a series of measures to control the spread. These measures may also have an impact on the epdemic patterns of other infectious diseases. This study utilized the ITS-ARIMA model to assess the impact of COVID-19 on gonorrhea and predict its epidemic trend, providing valuable insights for effective prevention and control strategies against gonorrhea.

In this study, an overal decline in gonorrhea cases was observed (AAPC = -3.5), followed by a slight reduction from 2018 to 2022 (APC = -5.6). The immediate drop in testing and notifications during the introduction of COVID-19 restrictions could be attributed to various factors, including changes in healthcare services, alterations in sexual practices, and reduced opportunities for disease transmission through international travel. The sustained decrease might indicate a lasting change in disease transmission, considering the increasing gonorrhea cases before the pandemic [22]. The estimated cumulative effect of the COVID-19 outbreak on the decline in gonorrhea case notifications in Fig. 6 indicated that despite a later-stage increase, the overall trend remained downward. Sensitivity analysis, comparing the ITS-ARIMA and BSTS models, consistently supported the effectiveness of the former, with a smaller MAPE (16.45% vs. 22.48%). The ITS-ARIMA model outperforms the BSTS model in simulating the gonorrhea epidemic trend and assessing prevention and intervention effectiveness. The superior predictive performance of the ITS-ARIMA model in this study may be attributed to the data type. Future research could explore alternative predictive models such as long short term memory neural network (LSTM) [23], Big Data Analytics Methods [24], or a hybrid of LSTM-ARIMA [25].

Since the implementation of COVID-19 restrictive measures in January 2020, the reported cases of gonorrhea have been notably affected, primarily due to a reduction in screening frequency. In the initial months of 2020, the positive detection rate for gonorrhea infections declined, aligning with findings from an Australian study [25]. Several factors contribute to this trend: First, social distancing and mobility restrictions likely reduced the risk associated with potential sexual behavior. Second, decreased access to screening occurred as in-person medical services. Third, healthcare personnel, including those specializing in gonorrhea management, were reassigned to address the pressing needs of the COVID-19 pandemic. Fourth, shortages in laboratory supplies may have hindered timely testing and reporting. Lastly, the severe phase of the COVID-19 pandemic may have resulted in reporting delays and missed cases in the gonorrhea data. Our research substantiated the underreporting of potential gonorrhea cases in China during the COVID-19 pandemic. However, exploring this topic is intricate due to various factors, including potential overlapping effects of other interventions or policies. The direct impact of COVID-19 control measures on public gatherings and access to public places could confound the normal transmission patterns of gonorrhea. Simultaneously, these measures may have deterred individuals from seeking resources for gonorrhea testing. China’s experience has shown that COVID-19 lockdowns resulted in insufficient and delayed opportunities for individuals with HIV/AIDS and tuberculosis to receive diagnosis and testing [26, 27]. The gonorrhea cases varied at different stages of the pandemic response, linked to differing levels of containment and closure policies [28]. These observations collectively suggested that gonorrhea may have continued to spread throughout the COVID-19 pandemic, albeit possibly at a lower transmission rate. Moreover, it is crucial to consider the lag effect of intervention measures, including delays in medical treatment, detection, and treatment of gonorrhea patients, along with suboptimal management. These factors not only worsen the severity of the disease but also heighten the risk of gonorrhea transmission. Currently, prioritizing access to care for individuals with gonorrhea and encouraging preventive measures among high-risk populations are crucial steps to mitigate the potential for a sharp resurgence in gonorrhea cases. Continuous nursing efforts, including telemedicine, offer an uninterrupted avenue for comprehensive gonorrhea care, ensuring detection, treatment, and prevention are not compromised during this period.

Gonorrhea, a prevalent venereal disease globally, exhibits seasonal variations in incidence. Typically, the incidence peaks in summer and autumn, while the trough in spring and winter. The autumn surge in gonorrhea cases may be linked to hormonal influences that promote increased sexual activity. Research has revealed elevated testosterone levels in the autumn, peaking in October, correlating with heightened sexual activity [29, 30]. The ITS-ARIMA model is effective in mitigating seasonal effects when modelling gonorrhea incidence. Globally, the prevalence of gonorrhea is most pronounced in Europe, the Americas, and some African countries [31]. The gonorrhea incidence is the highest among the poor, sexually active people, adolescents, blacks, less educated people, and unmarried people, who play a role in spreading gonorrhea [32]. Efforts to combat gonorrhea should focus on improving the detection of gonococcal resistance and raising the criteria for diagnosis and treatment of gonorrhea patients. Increasing public awareness of self-protection measures is crucial for reducing incidence. Additionally, prioritizing early detection and prompt treatment remains fundamental in the ongoing battle against gonorrhea.

Limitations should be mentioned in this study. First, passive monitoring systems may inevitably lead to underreporting, under-diagnosis, or delayed reporting. Second, our analysis utilized the national monthly gonorrhea incidence data, making it challenging to incorporate influencing factors such as air quality and weather conditions at a natinal level. Also, certain socio-economic indicators are only reported on a quarterly basis in China, further integration of these influencing factors was excluded. Lastly, policy landscapes are dynamic and subject to rapid changes, underscoring the need for further research to enhance our understanding of the situation and its complexities.

## Conclusion

ITS serves as a valuable tool for gauging intervention effectiveness, providing flexibility in modeling various impacts. The ITS-ARIMA model, adept at assessing trends and adjusting for serial correlation and seasonal impact, serves to evaluate the influence of COVID-19 interventions on gonorrhea. Moreover, the ITS-ARIMA model proves instrumental in predicting the trajectory of gonorrhea incidence, offering a robust foundation for effective prevention and control strategies. Our findings suggested a sustained decline in gonorrhea incidence since the COVID-19 measures began, not solely attributed to reduced testing but influenced by factors like prolonged social distancing and reduced high-risk behavior. As restrictions ease, prioritizing care for gonorrhea patients and promoting preventive measures are crucial to prevent a potential resurgence.

### Supplementary Information

**Additional file 1.**

## Data Availability

Gonorrhea data comes from China Center for Prevention and Control. The dataset supporting the conclusions of this article is included within its Supplementary file. The material was publicly available (http://www.nhc.gov.cn/jkj/s2907/new_list.shtml).
